# A comparison of sex, morphology, physiology and behavior of black-capped chickadees trapped using two common capture methods

**DOI:** 10.7717/peerj.10037

**Published:** 2020-09-22

**Authors:** Sara M. Burns, Frances Bonier

**Affiliations:** Department of Biology, Queen’s University, Kingston, ON, Canada

**Keywords:** Sampling bias, Field biology, Animal personality, Glucocorticoids, Corticosterone, Neophobia, Risk taking, Ornithology, Trapping methods

## Abstract

Many biological studies require the capture of individuals for sampling, for example for measurement of morphological or physiological traits, or for marking individuals for later observations. Capture methods employed often vary both within and between studies, and these differing methods could be more or less effective in capture of different individuals based on their morphology or behavior. If individuals that are prone to capture by the selected method differ with respect to traits of interest, such sampling bias could generate misleading or simply inaccurate results. The selection of capture methods could introduce two different forms of sampling bias, with the individuals that are sampled differing from the population at large or with individuals sampled via one method differing from individuals that could be sampled using a different method. We investigated this latter form of sampling bias by comparing individual birds sampled using two common capture techniques. We caught free-ranging black-capped chickadees (*Poecile atricapillus*) using walk-in traps baited with seed and mist nets paired with playback of an audio stimulus (conspecific mobbing calls). We measured 18 traits that we expect might vary among birds that are trappable by these differing methods—one that targets birds that are food motivated and potentially less neophobic and another that targets birds that respond readily to a perceived predation risk. We found no differences in the sex, morphology, initial and stress-induced corticosterone concentrations, behavioral response to a novel object, or behavioral response to a predator between individuals captured by these two methods. Individual variation in the behavioral response to a novel object was greater among birds caught by mist nets, suggesting this method might provide a sample that better reflects population-level individual variation. We do not know if the birds caught by these two methods provide a representative sample of the population at large, but can conclude that selection of either of these two common capture methods can similarly sample mean trait values of a population of interest. To accurately assess individual variation, particularly in behavior, mist nets might be preferable.

## Introduction

Almost all biological studies require the collection of data from a subset of individuals, from which inferences are made about a broader population. Implicit to this approach is an assumption that the individuals we sample are a random and representative subset of this population of interest (Biro, 2013; Fowler, Cohen & Jarvis, 2013). However, some of the sampling methods we use in data collection might introduce bias. For example, in studies of free-ranging animals, we might inadvertently skew our samples towards individuals that we can capture by a particular method because they exhibit certain behaviors (Garamszegi, Eens & Török, 2009; Biro, 2013; Stuber et al., 2013; Biro & Sampson, 2015). These individuals might represent a non-random sample in terms of their life history, morphology, physiology and/or behavior.

Sampling bias might be introduced by capture method in at least two different ways. First, individuals that are captured might differ from those that are not captured. Second, different capture techniques might more readily trap individuals that differ with respect to their morphology, physiology, behavior, or other traits. The first form of bias might be driven by traits that influence an individual’s propensity to be captured at all (i.e., its trappability). Specifically, individuals that are bolder, more active and less neophobic might often be over-represented in a sampled population relative to the broader population (Biro & Dingemanse, 2009). Evidence of this form of capture bias has been found in studies of numerous taxa, including fish (Biro & Post, 2008; Wilson et al., 2011), lizards (Carter et al., 2012), mammals (Tuyttens et al., 1999; Réale et al., 2000; Boon, Réale & Boutin, 2008), birds (Garamszegi, Eens & Török, 2009; Stuber et al., 2013; Camacho, Canal & Potti, 2017) and invertebrates (Biro & Sampson, 2015; Niemelä, Lattenkamp & Dingemanse, 2015). In some studies, trappability itself has been used as a measure of personality, with willingness to enter a trap interpreted as an indicator of propensity for risk taking (Réale et al., 2000; Boon, Réale & Boutin, 2008, but see Brehm & Mortelliti, 2018).

We have good evidence that probability of capture is not equal for all individuals in a population, and that the individuals that can be captured sometimes differ from those that cannot (examples reviewed above). However, when studies require the capture of individuals, for instance to measure morphological or physiological traits, we must implement some method of capture. Decisions about which capture method to use are often made based on convenience or convention, without knowledge of whether or not different capture methods select for different individuals. For example, field ornithologists commonly employ several capture methods, such as mist nets, food-baited traps, or nest-box traps (Elekonich & Wingfield, 2000; McGlothlin et al., 2008; Bonier, Moore & Robertson, 2011; DeVries, Winters & Jawor, 2012), sometimes without evaluating if traits of interest differ among individuals captured by the selected method(s) versus alternative available methods. Prior studies have provided ample evidence that capture methods can differ in effectiveness, as well as the species, age, sex, size and condition of captured individuals (Boonstra & Krebs, 1978; Weatherhead & Greenwood, 1981; Spence & Niemelä, 1994; Bauchau & Van Noordwijk, 1995; Gorney, Clark & Yom-Tov, 1999; Anthony et al., 2005; Burger et al., 2009; Stokes, 2013). Fewer studies have compared personality-related traits of individuals caught by different methods (Wilson et al., 2011; Michelangeli, Wong & Chapple, 2016). To our knowledge, all prior studies that have compared endocrine traits of individuals caught by different methods have tested for the effects of capture methods on hormone concentrations (e.g., due to initiation of a stress response), rather than testing for endocrine trait sampling bias due to capture method (Fletcher & Boonstra, 2006; Lynn & Porter, 2008; Angelier et al., 2010).

The propensity to be trapped by certain capture methods can be influenced by a number of individual traits, such as morphology, sex, physiology and behavior. For example, some obvious differences in morphology, such as body size, could bias which individuals are captured because of trap design (e.g., size-selective trapping methods). An individual’s condition and prospects for future reproduction might influence risk taking behavior, with individuals with greater future fitness prospects being more risk averse, and individuals in poor condition being more willing to take risks, particularly to access food (Clark, 1994; Killen, Marras & McKenzie, 2011; Nicolaus et al., 2012). Sex can also influence behavior in many contexts, including territoriality, offspring defense, and foraging (Regelmann & Curio, 1986; Clarke et al., 1998; Ruckstuhl, 1998; Smith, Bronstein & Papaj, 2019), and thus trappability might also differ among males and females. Endocrine traits (e.g., circulating concentrations, responsiveness of endocrine axes, etc.) can vary considerably among individuals often in ways that correlate with, and potentially also regulate, individual behavior or personality (Koolhaas et al., 1999; Cockrem, 2007; Williams, 2008; Carere, Caramaschi & Fawcett, 2010; Baugh et al., 2013; Cockrem, 2013). These endocrine differences might be associated with individual variation in trappability. For example, glucocorticoid hormones differ with respect to, and regulate, an individual’s metabolic rate, foraging behavior and life history stage (Landys, Ramenofsky & Wingfield, 2006; Lohmus, Sundström & Moore, 2006; Wada, 2008; Wack et al., 2012; Jimeno, Hau & Verhulst, 2017), and thus might differ among individuals caught by methods using food versus broadcast of sounds (e.g., conspecific song or mobbing calls) as lures. Hormone concentrations can also vary consistently among individuals (Schoenemann & Bonier, 2018), and can be associated with behavioral traits such as risk-taking, boldness and exploratory behavior (Koolhaas et al., 1999; Martins et al., 2007; Baugh et al., 2012; Baugh et al., 2013), all of which could influence an individual’s propensity to approach or investigate a trap. Finally, personality, by definition, predicts behavior across contexts, and might be one of the most important drivers of sampling bias (Biro & Dingemanse, 2009; Garamszegi, Eens & Török, 2009), with different traps potentially selecting for individuals that exhibit different behaviors.

Because of the variety of factors that could contribute to sampling bias, its occurrence could have a large impact on the outcomes of many biological studies, in some cases even resulting in skewed or inaccurate conclusions. Here, we test the hypothesis that capture method influences which individuals are sampled in wild populations by comparing multiple traits of free-ranging black-capped chickadees (*Poecile atricapillus*) captured using two different and common capture techniques: walk-in traps baited with food and mist-nets paired with broadcast of an audio recording of a conspecific anti-predator response.

We measured a suite of 18 traits including genetic sex, initial and capture stress-induced corticosterone, morphology (size, mass, fat, body condition) and foraging behavior at baseline and in the presence of two different stimuli (cues of predator presence and a novel object). We chose the two capture methods based on their routine use in field ornithological studies, and because of their potential to nonrandomly capture birds that differ from each other. Walk-in traps require that individuals enter a wire cage to forage, and so might select for more food motivated and less neophobic individuals, whereas mist nets are designed to be practically invisible to birds (with more or less success depending on setting and species), and so they select for individuals responsive to the paired lure(s), if any is used. In this study, pairing mist nets with playback of conspecific mobbing of a predator of adult chickadees should select for bolder individuals that are more responsive to perceived predation risks. As such, we predicted that individuals caught by these two methods might differ with respect to sex, morphology, physiology and/or behavior.

## Materials and Methods

### Study system

We sampled black-capped chickadees at 10 different sites on properties of the Queen’s University Biological Station (QUBS; near Elgin, Ontario; 44°34′N, 76°19′W) from October through December of 2014. Black-capped chickadees form flocks generally comprising 3–12 individuals and occupy a relatively stable winter territory (Foote et al., 2010), permitting sampling of multiple individuals at one site. Chickadees readily come in to human-provided food sources, such as bird feeders, and so can be captured with food-baited traps. Finally, black-capped chickadees and their relatives in the family Paridae (e.g., blue tits and great tits) are a common subject of a diverse array of biological studies involving capture and sampling.

### Capture and sampling

We captured birds between 07:00 and 13:00, starting no earlier than 1 h after sunrise to minimize the influence of diel variation in circulating hormone concentrations. We trapped at a given site for no more than 112 min within this time window (mean time from capture of first to last bird at a given site = 86 min). We used both capture methods at each site: walk-in (Potter) traps baited with black oil sunflower seeds and mist nets paired with playback of an audio recording of chickadees mobbing an eastern screech-owl (*Megascops asio*). Eastern screech-owls are a predator of adult black-capped chickadees, and their presence elicits an anti-predator behavioral response (Shedd, 1983; Foote et al., 2010). We put baited walk-in traps on platforms (~1.5 m off the ground) out at sites with their doors secured open for several days before sampling (mean = 13 days; range = 3–24 days) to allow birds to locate the seed and become accustomed to entering the traps to forage. This acclimation period should reduce the role of individual variation in neophobia in capture by this method. We sampled at nine sites on the same day, alternating the order of which capture method we used first between sites, and waiting a minimum of 30 minutes after capture of the last individual by the first method before beginning use of the second technique at the same site. At one site, we mist netted on the day after trapping via walk-in trap because of deteriorating weather conditions on the first day. Results do not differ if data from this site are excluded. When mist netting, we immediately turned off playback once an individual became entangled in the net. Playback was resumed only when one person could watch the net again to stop playback and extract the next captured bird. Audio playback never extended beyond 10 min of uninterrupted play (mean = 198 s; range = 10–600 s). Because chickadee flocks typically consist of 6–8 individuals (Smith, 1991), we limited sampling to the first three birds captured by each method, to ensure both methods could be used on each flock. This approach should also maximize our chance of detecting differences among birds caught by the two methods, if they exist, based on the assumption that the first few birds caught by each method are those that are most prone to capture by the method (if individuals vary in their trappability by these methods). Trapping sites were spaced to minimize flock territory overlap (mean distance = 8.8 km; range = 285 m to 21 km). We did not capture any individuals at more than one trapping site. We also did not capture any individual by more than one method, within each site.

Upon capture, we collected an initial blood sample into a heparinized microcapillary tube via puncture of the brachial vein (maximum 70 μL) with a 26-gauge needle as quickly as possible after the bird entered either capture device (i.e., the time when the door closed on the walk-in trap or the bird became entangled in the mist net). Three minutes post-capture is commonly used as a cut-off for measurement of baseline corticosterone (the primary glucocorticoid in birds) (Romero & Reed, 2005); however, we found a linear increase in ln-transformed corticosterone with sampling time (Fig. S1; linear model: *N* = 52, β = 0.006 ± 0.001, *p* < 0.001). Because of this relationship with sampling time, and differences in sampling time between methods (mean sampling time: walk-in trap = 135.2 s, mist net = 162.1 s; *t*-test: *t* = 2.27, df = 44.11, *p* = 0.03), we used the residuals of the linear regression of ln-transformed corticosterone on sampling time including samples collected past the 3-min time period (mean sample time = 148.12 s, range = 55–275 s) in subsequent analyses. Results do not differ if the 10 samples collected beyond 3 min post-capture are excluded from analyses, or if raw corticosterone concentrations, uncorrected for the relationship with sampling time, are used. The amount of time elapsed from initiating the capture attempt to the time the bird was captured was unrelated to corticosterone (linear model: β = −7.34 × 10^−5^ ± 7.79 × 10^−5^, *p* = 0.35), suggesting no effect of duration of disturbance due to our presence or audio playback at the trapping site.

We collected morphological measurements from each bird, including flattened wing chord using a wing ruler (±0.5 mm), tarsus length with calipers (±0.1 mm), and body mass with a Pesola spring scale (±0.5 g). We estimated a fat score for each individual by observing the amount of visible fat deposits in the furculum (inter-clavicular depression) using a scale from 0 (no visible fat) to 5 (excessive fat deposits) (Krementz & Pendleton, 1990). We calculated a scaled mass index (SMI) to estimate body condition of each individual using the slope of the least-squares regression of ln-transformed body mass (grams) on ln-transformed wing length (mm). We used wing length instead of tarsus length because it was more strongly correlated with body mass. We followed the Thorpe-Lleonart equation to estimate SMI: individual body mass × (population mean wing length/ individual wing length)^slope (Peig & Green, 2009). In this equation, the “slope” exponent is the scaled major axis slope, which is estimated as the slope from the least-squares regression divided by the correlation between body mass and wing length (Peig & Green, 2009). In our data, the correlation between body mass and wing length was 0.70, and the SMA slope was 2.12. The population mean wing length was 66.16mm.

We fit each bird with a uniquely numbered Canadian Wildlife Service aluminum band, a plastic colored leg band (Avinet, Dryden, NY, USA), and a passive integrative transponder (PIT) leg band (Eccel Technology, Leicester, UK). After we had obtained all measurements and banded birds, we placed them in an opaque, breathable cloth bag until 30 minutes after capture. We then collected a second blood sample for measurement of capture stress-induced corticosterone levels using the same collection method as described above. We failed to collect sufficient blood to measure initial or stress-induced corticosterone for a few birds (three initial, two stress-induced). We kept all blood samples cool by storing them on ice for transport to the laboratory at Queen’s University. All capture and handling methods were approved by the Queen’s University Animal Care Committee (protocol 2013-057) and banding was conducted under a Canadian Wildlife Service banding permit (permit 10771).

### Behavioral assays

We completed all behavioral trials during December 2014, between 08:00 and 12:00, a minimum of 22 days after capture (mean = 35 days; range = 22–49 days). We used radio frequency identification (RFID) readers mounted to bird feeders (Bird Feeder Reader Meter, IB Technology, Buckinghamshire, UK) at the feeding stations that had been established at the time of capture at each site. Birds at each site were allowed to habituate to the RFID feeder readers for a minimum of two full days (mean = 6; range = 2–8). We used RFID readers to measure activity at the feeder for three separate segments of the behavioral trials (details below). We set readers to record individual PIT tag numbers, time and date twice per second. We carried out trials over three consecutive days, with different segments of data collection occurring on separate days: a pre-trial control period, response to a predator model, and response to a novel object, with the type of stimulus (model predator or novel object) presented on the second or third days, alternating order between sites. Each trial lasted 2 h and all trials occurred at the same time of day within sites, allowing for direct comparison of behavior among trials. To assay risk-taking in response to a predation threat, we exposed each flock of chickadees to a small owl model paired with playback of an audio recording of an eastern screech-owl’s call. We used five different screech-owl recordings, all obtained from the Macaulay Library at the Cornell Lab of Ornithology. We played each audio recording from a FoxPro Scorpion X1B speaker (FOXPRO Inc., Lewistown, PA, USA) programed to play back the audio stimulus (5 min) interspersed with segments of silence of varying duration (range 3–13min). Each site received the same program of audio stimulus and silence, but we varied which of the five recordings was used. We checked volumes of each call with a sound meter to ensure all were played at 80–90 decibels at a distance of 1 m. This range reflects natural variation of screech-owl vocalizations as captured within the recordings we used, and playback volume was similarly varied across all sites. At each site, we placed the model predator and speaker in a tree 1–1.5 m from the RFID feeder reader. We then left the area for 2 h and used the RFID log to determine number of visits to the feeder in the presence of the predator stimulus. To assay willingness to visit the feeder in the presence of a novel object, we placed a red plastic drinking cup directly on top of the RFID feeders and left it in place for 2 h, again recording visits from the RFID log. Some of the initially captured birds were not recorded at RFID feeders during behavioral trials. Sample sizes are provided for each measurement (Table 1).

**Table 1:** Summary statistics and results of conditional inference tree analysis of several traits in black-capped chickadees caught by mist net or walk-in trap.

Trait	Sample size^1^	Mean value (std dev)		ctree *p*-value^2^	Levene’s *F. p*-value^3^
		Mist net	Walk-in trap		
Sex	28, 27	16F, 12M	12F, 15M	1.00	
Initial corticosterone^4^ (ng/ml)	25, 27	7.25 (3.18)	6.45 (3.08)	1.00	0.16, 0.69
Stress-induced corticosterone (ng/ml)	27, 26	30.5 (15.0)	30.7 (14.1)	1.00	<0.01, 0.98
Mass (g)	27, 27	11.0 (0.77)	11.2 (0.81)	1.00	0.21, 0.65
Tarsus length (mm)	28, 27	19.2 (0.58)	19.4 (0.58)	0.89	0.10, 0.75
Wing length (mm)	28, 27	65.9 (2.29)	66.5 (2.19)	1.00	0.01, 0.94
Scaled mass index (g)	27, 27	11.1 (0.69)	11.1 (0.53)	1.00	0.76, 0.39
Fat score (0–5)	28, 27	1.1 (0.48)	1.0 (0.69)	1.00	**4.18, 0.05**
Number of feeder trips, control trial^5^	18, 22	36.6 (37.5)	42.1 (46.9)	1.00	0.12, 0.73
Number of feeder trips, predator trial^5^	18, 22	24.9 (29.9)	19.8 (32.6)	1.00	0.14, 0.71
Number of feeder trips, novel object trial^5^	18, 22	30.9 (30.2)	28.0 (28.7)	1.00	0.01, 0.92
Mean duration of feeder trips, control trial (seconds)^5^	18, 22	1.2 (0.64)	1.0 (0.59)	1.00	0.79, 0.38
Mean duration of feeder trips, predator trial (seconds)^5^	18, 22	0.9 (0.45)	1.1 (0.79)	1.00	3.11, 0.09
Mean duration of feeder trips, novel object trial (seconds)^5^	18, 22	1.3 (0.75)	1.2 (0.77)	1.00	0.20, 0.66
Scaled change in number of trips, predator trial^6^	18, 21	0.23 (1.90)	−0.30 (0.90)	1.00	1.06, 0.31
Scaled change in number of trips, novel object trial^6^	18, 21	0.75 (1.75)	−0.29 (0.52)	0.24	**9.56, <0.01**
Scaled change in mean duration of trips, predator trial^7^	18, 21	−0.04 (0.65)	0.25 (1.09)	1.00	0.78. 0.38
Scaled change in mean duration of trips, novel object trial^7^	18, 21	0.33 (1.08)	0.16 (0.65)	1.00	0.98, 0.33

**Notes:**

1 Sample size is reported for mist nets and walk-in traps, respectively.

2 *p*-values are derived from conditional inference tree analysis using the command *ctree* in the *partykit* package in R.

3 *F* statistics and *p*-values derived from Levene’s tests comparing variances from the median among capture methods, degrees of freedom = 1.

4 Raw values for initial corticosterone are shown, but residual corticosterone, controlling for a relationship with sampling time, was analyzed. See main text for details.

5 Number and duration are raw values for the number of times an individual was logged as visiting the RFID feeder, or the mean duration of each trip, estimated as the total duration of time the bird was detected on the feeder perch divided by number of trips during each 2-h trial.

6 Scaled change in number of trips to the RFID feeder calculated for each individual as: (number of visits during the stimulus trial—number of visits during the pre-trial control period)/(number of visits during the pre-trial control period).

7 Scaled change in duration of time spent at the RFID feeder calculated for each individual as: (mean duration of trips during the stimulus trial—mean duration of trips during the pre-trial control period)/(mean duration of trips during the pre-trial control period).

Significant differences are indicated in bold font.

We assessed response to a predator and a novel object by tallying the total number of trips to the feeder and the mean duration of time spent at the feeder during each trip (total duration of time detected at the feeder in seconds, divided by number of trips) for each individual during each 2-h trial (pre-trial control, predator trial, novel object trial). If an individual was detected at the feeder during some but not all of the trials, we assigned them a value of zero for both behaviors (number and mean duration of visits) for the trials they when they were not detected, but we excluded individuals that were never detected at the feeder during any of the trials from the analyses. We analyzed foraging behavior using both raw values for each individual (number and mean duration of visits to the feeder during each trial) and by calculating the change in behavior relative to the control trial (i.e., (number or duration of visits during predator or novel object exposure—number or duration of visits during pre-trial control)/number or duration of visits during pre-trial control). With this calculation, a negative value represents a reduction of trips to or duration of time spent at the feeder, a positive value represents an increase, and a zero reflects no change. The magnitude of the value reflects the scale of the change relative to behavior in the absence of the stimuli. Importantly, with this calculation of change in behavior, values cannot be lower than −1, which represents individuals that were present during the pre-trial control but did not visit the feeder during the predator or novel object trial (number/duration of feeder visits = 0). One individual that visited the feeders during the novel object trial was excluded from these analyses because it did not visit the feeder during the pre-trial control (or predator trial), and so a scaled change in behavior could not be calculated. Results do not differ if the response to the stimulus is calculated as a simple difference without scaling by the control value (e.g., stimulus behavior–pre-trial control behavior). We did not assess repeatability of behaviors within individuals and so do not assume that foraging behaviors during the various trials reflect individual variation in personality. Previous estimates suggest that activity, response to novel objects, and exploratory behaviors have low to moderate repeatability in black-capped chickadees (Devost et al., 2016) and other bird species (Van Oers et al., 2004; Bell, Hankison & Laskowski, 2009; Garamszegi et al., 2015; Audet, Ducatez & Lefebvre, 2016; Holtmann, Lagisz & Nakagawa, 2017).

### Hormone assay

We centrifuged blood samples for 5 min at 6,000 rpm within 6 h of collection, which is well within the timing during which hormone concentrations remain stable at cool temperatures (Khonmee et al., 2019). We then preserved plasma and red blood cells separately at −20 °C until further processing. We measured total plasma corticosterone using an enzyme immunoassay (EIA) with a detection limit of approximately 30 pg/mL (Lot No. 0457099; Cayman Chemical Co., Ann Arbor, MI, USA). No samples fell below this detection limit. Prior to assaying experimental samples, we validated the assay for use in this species by assaying a serial dilution of pooled plasma collected from multiple black-capped chickadees, sampled during the same life history stage, as well as a serial dilution of pooled plasma that was spiked with corticosterone to increase concentration by 10 ng/ml. Both serial dilution curves ran parallel to the assay standard curve. We assayed 7.5 μL of each plasma sample from focal birds in duplicate after dilution (1:16, using steroid buffer) on three plates following supplier instructions. We ran eight known-concentration standards, also in duplicate, on each plate to determine intra and inter-plate variation. The coefficient of variation based on these replicate standards was 5.3% between plates and averaged 3.0% within plates. Baseline corticosterone concentrations in this population during the non-breeding season have been estimated to be somewhat repeatable (*r* = 0.26; Montreuil-Spencer et al., 2019). We have not estimated repeatability of stress-induced corticosterone concentrations for this population, but they are generally more repeatable than baseline concentrations (Schoenemann & Bonier, 2018). As such, individual variation in corticosterone concentrations is expected to reflect both flexible and consistent differences among individuals.

### Molecular sexing

To differentiate male and female black-capped chickadees, which are not highly dimorphic in morphology or plumage, particularly outside of breeding, we extracted DNA from red blood cells using Qiagen DNeasy Blood and Tissue Kits following the supplier’s protocol for nucleated blood cells (Qiagen Inc., Toronto, ON, Canada). We used molecular sexing techniques as described by Griffiths et al. (1998). After separation of PCR products by electrophoresis, we identified females by the presence of two bands (WZ) and males by the presence of a single band (ZZ). As quality control measures, we included samples from birds of known sex as well as negative controls in all PCRs, which always produced expected results.

### Statistical analysis

We completed all analyses using R (version 4.0.0), and all raw data and code for the main analysis have been uploaded to Open Science Framework (available at: https://osf.io/zsjme/). To determine if individuals captured by the two methods differ for any of the traits we measured, we used conditional inference tree analysis using the *ctree* function in the package partykit (Hothorn & Zeileis, 2015). Conditional inference tree analyses are a form of non-parametric regression tree analysis that uses recursive partitioning to identify factors that predict variation in a response variable (Hothorn, Hornik & Zeileis, 2006; Hothorn & Zeileis, 2015). We included all of the trait measurements as candidate predictor variables (Table 1) and method of capture (walk-in trap or mist net) as the response variable. Results do not differ if we instead analyze each trait separately as a response variable in a linear mixed-effect model with capture method as the sole fixed effect and capture site, order of use of capture methods and/or order of stimulus presentation (for behavioral data) as random effects. Conditional inference tree and other decision tree approaches are increasingly being used in ecological research (Johnstone, Lill & Reina, 2014; Maslo et al., 2016; Burke et al., 2020; d’Entremont et al., 2020), and can be preferable to traditional regression methods as they can allow for a more conservative, refined and holistic approach to identifying differences among groups (Cutler et al., 2007; Müller, Schröder & Müller, 2009; Blank & Blaustein, 2014). Conditional inference trees are also powerful because of their ability to simultaneously assess categorical and continuous variables (including data with non-normal distributions) and to accommodate missing values (Hothorn, Hornik & Zeileis, 2006).

We conducted a follow-up analysis on a subset of our data to determine if the birds that were most readily caught by each method differed from each other for any of the traits that we measured. For this analysis, we repeated the conditional inference tree analyses as described above, but limited to the first three individuals caught at each site, based on the assumption that these first-caught birds were the most trappable. This follow-up analysis suffers from the limitations of a reduced sample size (*N* = 30) and a lack of pairing of methods within sites, but could be informative, particularly if results differed from those of our main analysis.

Some capture methods might select for a subset of the individuals captured by other methods with a narrower range of trait values, which could result in similar means but different variances among groups. As such, in addition to analyses of mean trait values, we also compared variances among individuals caught by the two capture methods using separate modified Levene’s tests of homogeneity of variances from the trait median, which is robust to departures from normality (Carroll & Schneider, 1985).

## Results

We captured a total of 55 birds at 10 different sites: 28 in mist nets and 27 in walk-in traps. Mean time from initiating the capture effort to capture of birds did not differ between the two methods, although it tended to be faster for walk-in traps (walk-in trap = 9.3 ± 1.7 min (mean ± SE elapsed time), *N* = 27; mist nets = 15.3 ± 3.0 min, *N* = 28; *t* = 1.68, df = 53, *p* = 0.10). Mean time from initiating the capture effort to capture of birds also did not differ with the order of method used at each site (first method = 12.4 ± 2.4 min, *N* = 30; second method = 12.3 ± 2.8 min, *N* = 25; *t* = 0.03, df = 53, *p* = 0.98). We collected behavioral data from 40 of the 55 PIT-tagged individuals.

**Figure 1:**
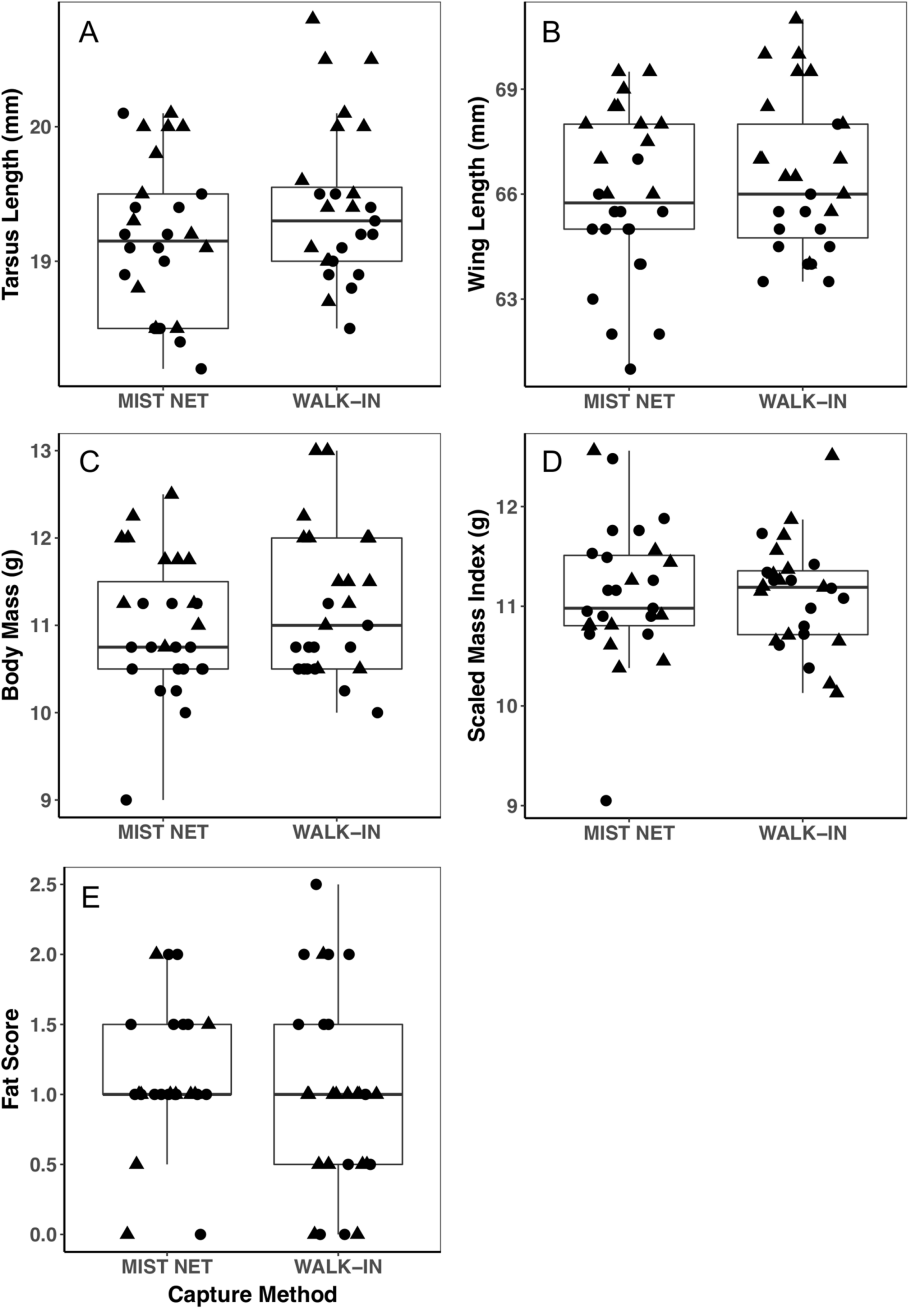
Morphological measurements of black-capped chickadees did not differ between individuals captured in mist nets or walk-in traps. Panels show tarsus length (A), wing length (B), body mass (C), scaled mass index (D), and fat score (E). Points represent individual measurements (circles = female, triangles = male) and are jittered along the *x*-axis for ease of visualization. Horizontal lines in each boxplot represent the median, with the extent of boxes representing the 25–75th percentile of the data, and whiskers representing 1.5 inter-quartile ranges. See Table 1 for statistical support.

**Figure 2:**
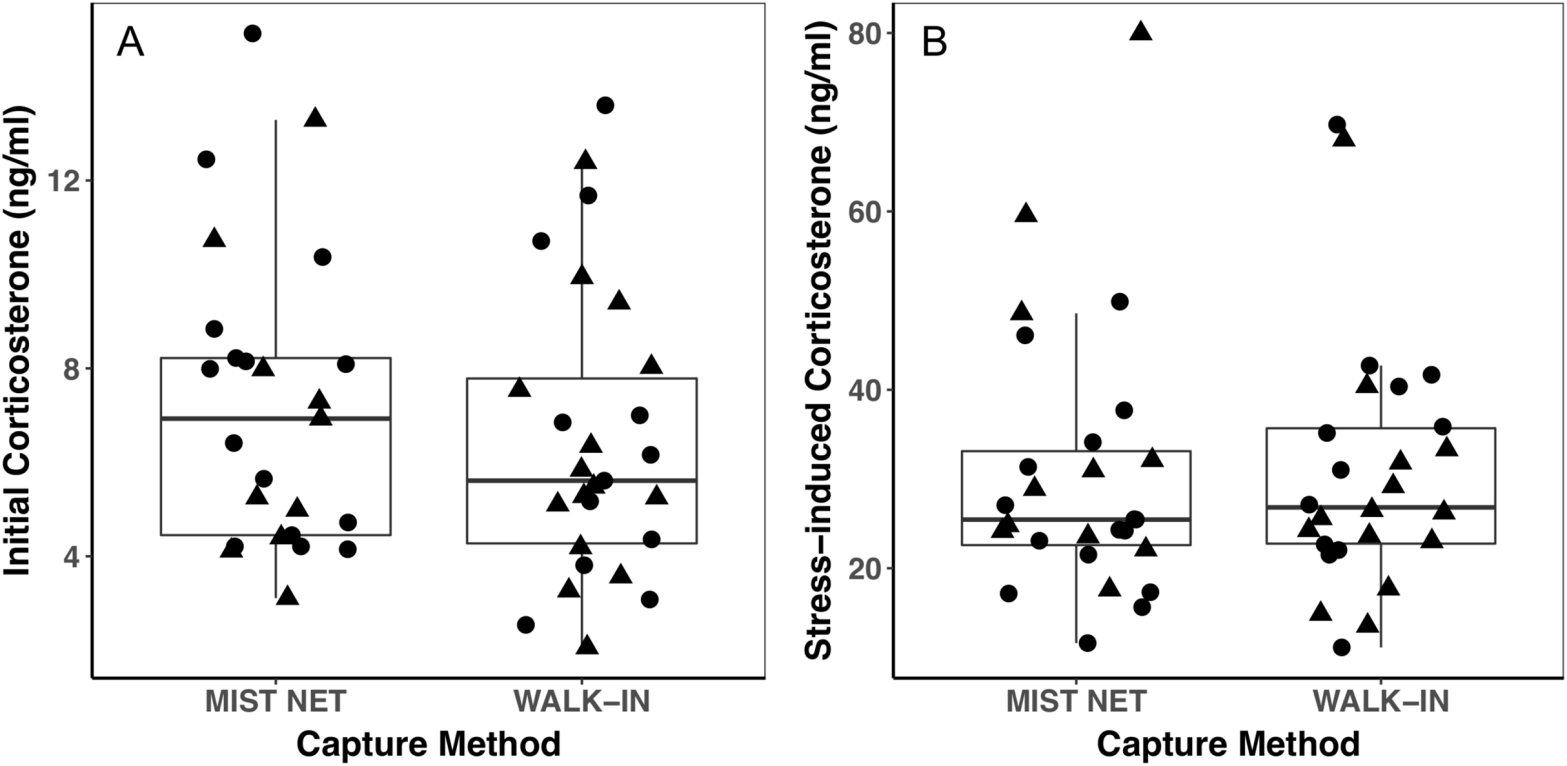
Corticosterone concentrations did not differ among black-capped chickadees captured in mist nets or walk-in traps. Points represent individual measurements (circles = female, triangles = male) and are jittered along the *x*-axis for ease of visualization. (A) shows initial corticosterone measured as quickly as possible following capture, and (B) shows corticosterone measured 30 min after capture. Analyses of initial corticosterone controlled for timing of initial sample, but uncorrected values are shown here. Horizontal lines in each boxplot represent the median, with the extent of boxes representing the 25–75th percentile of the data, and whiskers representing 1.5 inter-quartile ranges. See Table 1 for statistical support.

**Figure 3:**
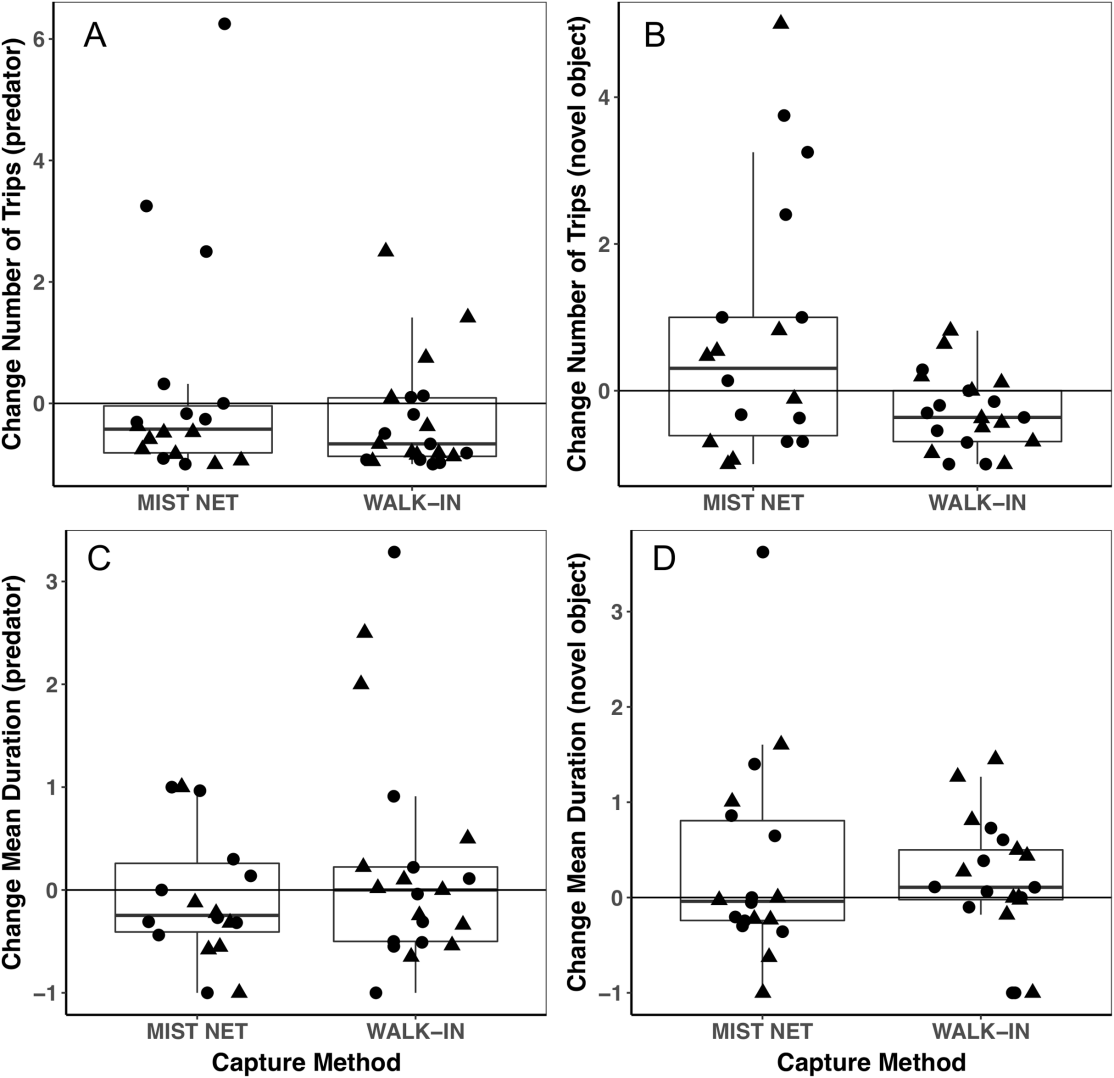
Behavioral responses to a model predator (A and C) and novel object (B and D) did not differ among black-capped chickadees captured in mist nets or walk-in traps. Points represent individual measurements (circles = female, triangles = male) and are jittered along the *x*-axis for ease of visualization. (A and B) show change in the number of trips recorded to a bird feeder equipped with a radio-frequency identification (RFID) receiver, scaled relative to the number of trips each individual made during a control trial of the same length (see main text for details). (C and D) show change in the mean duration of trips to the RFID bird feeder, scaled relative to the mean duration of trips to the feeder during the control trial. Negative values represent a reduction of trips to or duration of time spent at the feeder, positive values represent an increase, and zeroes reflect no change, relative to behavior during a control trial. Horizontal lines in each boxplot represent the median, with the extent of boxes representing the 25–75th percentile of the data, and whiskers representing 1.5 inter-quartile ranges. See Table 1 for statistical support.

Individuals caught by walk-in traps and mist nets did not differ in sex (16F, 12M mist net; 12F, 15M walk-in trap) or any of the morphological (Fig. 1), endocrine (Fig. 2), or behavioral (Fig. 3) traits we measured. None of the traits were identified as covariates of capture method (conditional inference tree analysis, all *p* > 0.24; Table 1). Results were similar when we repeated analyses on only the first three individuals captured at each site, with even less evidence for differences among birds caught by the two methods (conditional inference tree analysis, all *p* > 0.72; Table S1).

Trait variances from the median were similar for endocrine traits (Fig. 2) and for foraging behavior during the different trials (modified Levene’s test, all *p* > 0.08; Table 1). Among morphological traits (Fig. 1), there was some evidence of greater variance in fat score among birds caught in walk-in traps relative to mist-netted birds (*F* = 4.18, df = 1, *p* = 0.05), but variances for all other morphological traits were similar (all *p* > 0.38). Variances of changes in rates and duration of visits to the RFID feeder during the predator trial were similar among birds caught by the two capture methods (Figs. 3A and 3C; all *p* > 0.30). Behavioral responses to the novel object trial were more variable among birds that were mist-netted relative to those caught in walk-in traps in terms of change in number of trips to the feeder (Fig. 3B; *F* = 9.56, df = 1, *p* = 0.004), but the variances of change in mean duration of trips was similar among groups (Fig. 3D; *F* = 0.78, df = 1, *p* = 0.38).

## Discussion

Using different capture methods has the potential to introduce sampling bias that could affect study outcomes. Here, we compared multiple traits of black-capped chickadees caught using two common capture techniques: mist nets paired with playback of an audio stimulus and walk-in traps baited with seed. The range of traits that we measured encompasses many of the traits that we expect to relate to trappability by these two methods. Despite this, we did not find evidence of differences in the sex, morphology (Fig. 1), corticosterone concentrations (Fig. 2), or behavior (Fig. 3) of individuals captured by the two methods. Although mean trait values did not provide evidence of capture bias, trait variances differed for one morphological measure (fat score, Fig. 1E) and one estimate of behavioral response to a novel object (Fig. 3B).

We cannot assess the degree to which our sample of captured birds represents the broader population. Bias between individuals that are captured compared to those that are not has been the focus of several studies and reviews (Biro & Dingemanse, 2009; Garamszegi, Eens & Török, 2009; Biro, 2013; Stuber et al., 2013; Biro & Sampson, 2015; Simons et al., 2015; Camacho, Canal & Potti, 2017), and represents a potential limitation for studies that require capture. Birds caught by both methods could be those in the population that generally exhibit higher levels of risk-taking, which has been found to predict trappability in other species (Garamszegi, Eens & Török, 2009; Gabriel & Black, 2010). We expected that bolder birds might approach the predator and conspecific mobbing cues that we paired with the mist nets, whereas more food motivated and less neophobic birds might enter the walk-in trap (although the habituation period we provided should reduce the role of neophobia). Depending on how they are defined and measured, boldness or aggression are sometimes correlated with neophobia, with bolder and more aggressive individuals also being less neophobic (Drent, Verbeek & Boon, 1996; Koolhaas et al., 1999; Sih, Bell & Johnson, 2004; but see, Audet, Ducatez & Lefebvre, 2016), but the relationship between boldness and neophobia is complex, variable, and dependent on context (Réale et al., 2007; Greggor, Thornton & Clayton, 2015; Biondi et al., 2020). Evidence in black-capped chickadees does not point to correlations between several personality-related traits, nor a relationship between those traits and social dominance (Devost et al., 2016), although subordinate chickadees exhibit higher risk-taking behavior than dominants in some contexts, including responses to predators and during foraging (Zanette & Ratcliffe, 1994; Ratcliffe, Mennill & Schubert, 2007). Both capture methods could have targeted birds that are bolder, less risk averse and less neophobic than birds that were not caught.

Although we did not identify any differences in mean trait values among birds captured in mist nets and walk-in traps, they could differ in ways that we did not measure. One important aspect of flocking species like the black-capped chickadee is their dominance hierarchy, which we did not assess. Individual differences in social rank can influence risk-taking behavior (Schneider, 1984; Hegner, 1985; Zanette & Ratcliffe, 1994; Ratcliffe, Mennill & Schubert, 2007; An et al., 2011), and so could affect both trappability and response to the model predator or novel object. If there were differences in dominance status between birds captured by the two techniques, we would expect to have detected differences in sex, morphology, body condition, or behavior, because dominance is often associated with these traits (Ficken, Weise & Popp, 1990; Smith, 1991; Van Oort et al., 2007; Ratcliffe, Mennill & Schubert, 2007; Fox et al., 2009; but see Devost et al., 2016).

We found a large degree of individual variation in behavioral responses to the two stimuli, most notably among mist-netted birds in response to the novel object (Fig. 3B), although mean responses did not differ. The difference in variances that we saw suggest that mist nets might capture a broader range of birds, perhaps better representing true population-level individual variation in behavior. Individuals might change, or not change, their behavior in response to the stimuli we presented for several reasons. In chickadees, we expect dominance hierarchies could influence the direction of response to the stimuli, with lower-ranked individuals being more likely to come in to the feeders during stimulus presentation, while dominants are avoiding the perceived threat (Ficken, Weise & Popp, 1990; Zanette & Ratcliffe, 1994; Ratcliffe, Mennill & Schubert, 2007). In contrast, in the absence of these stimuli, subordinates might have their access to the feeder limited by dominants during the control period (Ficken, Weise & Popp, 1990; Ratcliffe, Mennill & Schubert, 2007). Interestingly, six individuals, all caught in mist nets, more than doubled their visits to the feeder in the presence of the novel object, relative to the control period (scaled change in number of trips ≥1), which drives the difference in variances that we found (Fig. 3B). These six individuals might have been subordinates that were excluded from the feeder during the control trial and took advantage of having greater opportunity to forage during the novel object trial, when dominants might have perceived the risk of foraging as too high. If differences in dominance status do explain this difference, this suggests that subordinates are less likely to be captured in walk-in traps than in mist nets. In contrast, five individuals (two caught in walk-in traps, three in mist nets) visited the feeder more than twice as often during the predator presentation as during the control period (Fig. 3A), which does not agree with a dominance hierarchy related interpretation. An additional implication of the high degree of variation we found in behavioral responses is that it reduces our power to detect small differences among the capture method groups, particularly given our relatively small sample sizes.

Our method of data collection cannot distinguish among reasons for individuals’ visits to the feeder. Chickadees mob predators by landing close to them and alarm calling (Shedd, 1983; Smith, 1991). We could not determine if birds perched on the RFID feeder as part of a mobbing response, to investigate the stimulus, to forage, or for some other reason. Despite these limitations in interpreting the behavioral data, we can conclude that mean frequencies and durations of visits to the feeder and mean changes in response to the stimuli did not differ among individuals caught by the two methods, although variances differed for one behavioral measure. Without further information, we cannot conclude that risk aversion does not differ among birds caught by the two methods. Increasing sample size, as well as pairing direct observations and data on dominance status with automated techniques would allow for more refined and robust assessments of individual behavior and potential capture bias.

## Conclusions

Experimental design is one of the most important components of any research effort, and choosing appropriate capture methods should be a priority. The type of capture techniques we use in studies of wild birds often depends on convention, logistics, the behavior of our study species, and the focal research question. All capture methods have the potential to introduce bias into our data. We have shown here that the use of either of two common capture methods—food-baited walk-in traps and mist nets paired with audio playback—do not introduce bias with respect to the sex, morphology, corticosterone concentrations, and, to some extent, behavior of black-capped chickadees. For one metric, individual variation in behavior was greater among birds caught by mist nets, suggesting this method might provide a sample that better reflects population-level individual variation. We expect these findings should apply to other similar songbirds, providing some reassurance that, when a study requires capture, either of these methods can be employed to similarly sample a population of interest when mean trait values are of primary interest; however, when individual variation is important to a research aim, mist nets may be preferable.

## Supplemental Information

10.7717/peerj.10037/supp-1 Supplemental Information 1 Initial corticosterone concentrations in black-capped chickadees increased with timing of sampling. Each point represents a measurement from an individual chickadee caught either by mist net or walk-in trap (circles = female, triangles = male). Time to sample (*x*-axis) is the time elapsed since capture (entanglement in the net or closing of the trap door) when the blood sample was collected. Analyses reported in the main text use residuals from this linear relationship (line = best fit from a linear model, *N* = 52, β = 0.006 ± 0.001, *p* < 0.001; shaded area = 95% confidence interval).

10.7717/peerj.10037/supp-2 Supplemental Information 2 Summary statistics and results of a complementary to the main conditional inference tree analysis of several traits in black-capped chickadees caught by mist net or walk-in trap. Subset analysis of only the first three birds caught at each site.

## Additional Information and Declarations

### Field Study Permissions

The following information was supplied relating to field study approvals (i.e., approving body and any reference numbers): Banding was conducted under a Canadian Wildlife Service banding permit (permit 10771).

### Data Availability

The following information was supplied regarding data availability: Raw data and R code are available at Open Science Framework: Bonier, Frances. 2020. “Capture Bias Black-Capped Chickadees.” OSF. July 18. osf.io/zsjme. The following recordings from the Macaulay Library at the Cornell Lab of Ornithology were used in behavioral trials: *Megascops asio* 20424, 20425, 20426, 20427, 61814, and are available from the Macaulay Library: https://macaulaylibrary.org/asset/20424 https://macaulaylibrary.org/asset/20425 https://macaulaylibrary.org/asset/20426 https://macaulaylibrary.org/asset/20427 https://macaulaylibrary.org/asset/61814.
