# Supplementary figures and images for: A comparison of sex, morphology, physiology and behavior of black-capped chickadees trapped using two common capture methods
Source: PeerJ. 2020 Sep 22;8:e10037. doi: 10.7717/peerj.10037 (PMC7518160; doi:10.7717/peerj.10037)

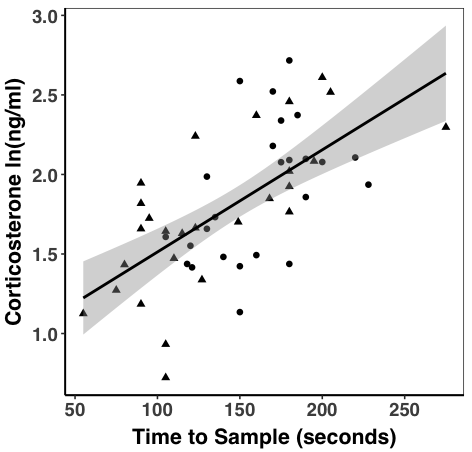

Supplement: Supplemental Information 1 — Each point represents a measurement from an individual chickadee caught either by mist net or walk-in trap (circles = female, triangles = male). Time to sample (x-axis) is the time elapsed since capture (entanglement in the net or closing of the trap door) when the blood sample was collected. Analyses reported in the main text use residuals from this linear relationship (line = best fit from a linear model, N = 52, β = 0.006 ± 0.001, p < 0.001; shaded area = 95% confidence interval). [file peerj-08-10037-s001.png]
