# Supplementary material for: A comparison of sex, morphology, physiology and behavior of black-capped chickadees trapped using two common capture methods
Source: PeerJ. 2020 Sep 22;8:e10037. doi: 10.7717/peerj.10037 (PMC7518160; doi:10.7717/peerj.10037)
Supplement: Supplemental Information 2 [file peerj-08-10037-s002.docx]

Table S1. Summary statistics and results of a complementary to the main conditional inference tree analysis of several traits in black-capped chickadees caught by mist net or walk-in trap. Subset analysis of only the first three birds caught at each site.

| Trait | Sample size^1^ | Mean value (std dev) | | ctree  *p*-value^2^ |
| --- | --- | --- | --- | --- |
|  |  | *Mist net* | *Walk-in trap* |  |
| Sex | 15, 15 | 8F, 7M | 7F, 8M | 1.00 |
| Initial corticosterone^3^ (ng/ml) | 13, 15 | 7.4 (3.5) | 6.9 (3.5) | 1.00 |
| Stress-induced corticosterone (ng/ml) | 15, 15 | 27.1 (10.7) | 33.9 (16.4) | 0.98 |
| Mass (g) | 14, 15 | 11.1 (0.95) | 11.2 (0.83) | 1.00 |
| Tarsus length (mm) | 15, 15 | 19.2 (0.56) | 19.5 (0.50) | 0.96 |
| Wing length (mm) | 15, 15 | 66.1 (2.15) | 66.2 (2.24) | 1.00 |
| Scaled mass index (g) | 14, 15 | 11.1 (0.80) | 11.2 (0.61) | 1.00 |
| Fat score (0-5) | 15, 15 | 1.0 (0.44) | 0.8 (0.70) | 1.00 |
| Number of feeder trips, control trial^4^ | 8, 13 | 33.3 (32.5) | 55 (57) | 1.00 |
| Number of feeder trips, predator trial^4^ | 8, 13 | 29.8 (35.8) | 22.9 (37.8) | 1.00 |
| Number of feeder trips, novel object trial^4^ | 8, 13 | 30.6 (29.8) | 32.8 (33.7) | 1.00 |
| Mean duration of feeder trips, control trial (seconds)^4^ | 8, 13 | 1.0 (0.68) | 1.1 (0.70) | 1.00 |
| Mean duration of feeder trips, predator trial (seconds)^4^ | 8, 13 | 1.0 (0.46) | 0.8 (0.80) | 1.00 |
| Mean duration of feeder trips, novel object trial (seconds)^4^ | 8, 13 | 0.9 (0.50) | 1.3 (0.82) | 0.99 |
| Scaled change in number of trips, predator trial^5^ | 8, 12 | 0.34 (2.4) | -0.16 (1.1) | 1.00 |
| Scaled change in number of trips, novel object trial^5^ | 8, 12 | 0.30 (1.4) | -0.31 (0.5) | 0.97 |
| Scaled change in mean duration of trips, predator trial^6^ | 8, 12 | 0.19 (0.76) | -0.30 (0.36) | 0.72 |
| Scaled change in mean duration of trips, novel object trial^6^ | 8, 12 | 0.01 (0.72) | 0.14 (0.57) | 1.00 |

^1^ Sample size is reported for mist nets and walk-in traps, respectively.

^2^ *p*-values are derived from conditional inference tree analysis using the command *ctree* in the *partykit* package in R.

^3^ Raw values for initial corticosterone are shown, but residual corticosterone, controlling for a relationship with sampling time, was analyzed. See main text for details.

^4^ Number and duration are raw values for the number of times an individual was logged as visiting the RFID feeder, or the mean duration of each trip, estimated as the total duration of time the bird was detected on the feeder perch divided by number of trips during each 2-hour trial.

^5^ Scaled change in number of trips to the RFID feeder calculated for each individual as: (number of visits during the stimulus trial – number of visits during the pre-trial control period) / (number of visits during the pre-trial control period).

^6^ Scaled change in duration of time spent at the RFID feeder calculated for each individual as: (mean duration of trips during the stimulus trial –mean duration of trips during the pre-trial control period) / (mean duration of trips during the pre-trial control period).
